# From Research to Practice: Enhancing Creativity Awareness in Education

**DOI:** 10.3390/bs16030440

**Published:** 2026-03-17

**Authors:** Iclal Can, William M. Bart

**Affiliations:** 1 Guidance and Psychological Counseling Program, Middle East Technical University Northern Cyprus Campus, 99738 Güzelyurt, Türkiye; 2 Department of Educational Psychology, University of Minnesota, Minneapolis, MN 55455, USA; bartx001@umn.edu

**Keywords:** creativity awareness, creativity enhancement, creative thinking, creativity

## Abstract

This selective integrative narrative review article examines creativity awareness as a critical cognitive state and highlights its intentional development in teaching and learning as part of essential 21st-century skill building. The article is organized around three interconnected themes: (1) Creativity as a 21st-century skill; (2) Exploring the journey to promoting creativity awareness in education: A multi-dimensional focus; and (3) Enhancing creativity awareness through research and science communication. Grounded in established and emerging research in the field, this review provides research-informed insights and practical recommendations for educators, researchers, and school counselors to recognize, appreciate, and promote creativity awareness in ways that contribute to more adaptive, innovative, and equitable educational environments.

## 1. Introduction

Creativity awareness is a cognitive state that has simple and complex aspects. More specifically, it is a multi-dimensional, context-sensitive psychological process encompassing cognitive, behavioral, and affective components. Regarding its simple aspect, cognitive awareness may be simply viewed as the perception of a creative act or entity that is both novel and functional, with novelty and utility widely regarded as prominent features of creativity (Diedrich et al., 2015). Although creativity awareness has been an implicit component of prior creativity research, the construct does not have a single explicitly articulated definition. In this selective integrative narrative review, creativity awareness is broadly defined as the capacity to recognize, appreciate, and respond to creative behavior within a particular environment.

To appreciate the complex aspects of creativity awareness, one needs to consider the environmental context of the creative act or entity. As an example, consider a scout for a professional soccer team viewing a youth soccer player, who is off balance, kicking a soccer ball in an unusual manner past a defender and a goalie into the corner of the opposing net for a winning score. The kick was novel as well as functional as it led to the winning score. The professional scout would likely have perceived this creative act as a demonstration of player creativity. The professional soccer scout very likely has creativity awareness as creativity among soccer players wins games.

In the context of soccer, creativity on the part of the participants, whether scouts or players or coaches, is highly valued. A soccer scout, whether professional or amateur, needs to be keenly aware of creativity among players. Not only must the scout have creativity awareness, but the scout acts when perceiving creativity in soccer. The scout will dutifully prepare a report on the creative act that the scout witnessed. In addition to preparing a report on player creativity, the scout will likely and enthusiastically advocate for the player manifesting creativity.

What a person does after perceiving creativity leads us to the complex aspects of creativity awareness. In the case of soccer, creativity is highly valued. As a result, after perceiving creativity in the behavior of a player, the scout not only reports on the player’s creativity but also advocates for the player manifesting the creativity. The professional scout very likely finds any player creativity to be a personally exciting and rewarding experience and likely hopes that the subsequent report and endorsement of the player manifesting the creativity in soccer will make a solid contribution to the future success of the soccer team employing the scout.

This example illustrates that creativity awareness is influenced by the environment in which it occurs. Environments might show variation regarding the extent to which creative behavior is recognized, appreciated, and supported. While physical environments can affect creativity (Lee & Lee, 2023; Richardson & Mishra, 2018), the environment fostering creativity is not limited to the physical space alone. Previous research has shown that the creativity-fostering environments encompass much more than physical settings including cultural norms and values, socio-emotional climate, and social interactions, perceptions, and expectations. Exploring how creativity awareness manifests itself in different and contrasting environments enables a deeper understanding of why it is appreciated and supported in one context and not valued or ignored in others. What makes this process even more complex is the dynamic interplay among these environmental factors and individual variables such as personality.

The sport of soccer is an environment in which creativity awareness is highly valued and supported, because creativity leads to team improvement and success. Unfortunately, creativity awareness is not highly valued and supported in all environments. Consider the school environment as one such example.

Beghetto (2019, 2021) recognized that school classrooms are marked by “sameness” in which similar students are presented with similar instruction to attain similar goals. Classrooms tend to be highly regimented with students being subjected to frequent evaluation. Both teachers and administrators are often under pressure to have students attain predetermined achievement goals. School classrooms tend to be difficult locations to be supportive of creativity awareness among teachers, students, and administrators.

However, Beghetto does identify some ways in which teachers can be creative. Beghetto contends that teachers can manifest creativity in their teaching. Teachers can teach about creativity by teaching how important ideas and events came to be. Teachers can teach for creativity by having students approach issues and topics from different perspectives. Teachers can teach with creativity by exploring topics in the academic lessons in greater depth that seem to interest students. Students can engage in creative learning by engaging in “real-world problem-solving projects” and projects based on ill-defined problems (Beghetto, 2019, p. 600). These approaches are discussed further in subsequent sections of this review, which draws on a broader body of research and policy publications on creative teaching and creativity awareness.

One interesting concept cited by Beghetto (2021) that warrants due consideration in this exposition of creativity awareness is the concept of creativity confidence. That concept is defined as “people’s belief that they can successfully think and act in creative ways” (Beghetto, 2021, p. 235; Karwowski & Beghetto, 2019). This belief can “play an important role in transforming creative potential into creative achievement” (Beghetto, 2021, p. 235).

Although Beghetto indicates how creativity can occur in the school classroom environment, the school environment tends not to be as supportive of creativity awareness as the soccer context described earlier. Teachers who have creativity awareness are neither expected nor required to report on creative acts or events among students or staff. They are also neither expected nor required to advocate for the students or staff who manifest creative behavior. School environments tolerate some creative behavior among teachers and students but are rarely influenced by teachers and students who have creativity awareness.

The sport of soccer and the school are but two environments where creativity awareness can occur. To evaluate the level of creativity awareness of an individual, one needs to consider the environment of an individual. Does the environment of the individual allow the individual to be aware of creative behavior? Does the environment encourage the individual with creativity awareness to report creative behavior? Does the environment change or even improve as the result of advocacy for creativity by the individual with creativity awareness? These are some major questions that may be asked in determining whether an environment is a fine setting for an individual with creativity awareness.

While creative teaching processes may foster student learning, motivation, and engagement, they may not automatically result in creativity outcomes and creativity awareness. Creativity awareness emerges from a dynamic, overlapping relationship among multiple factors including instructional practices, classroom climate, personality factors, student agency, and the opportunities educators and parents create for students to experiment with creative ideas. This complexity highlights the need for a clearer understanding of what creativity awareness is, how individuals can be guided to recognize, value, and respond to creative behaviors, and how learning environments can be intentionally designed to support creativity awareness.

In this review paper, we present and discuss creative awareness as an important cognitive state that can be enhanced in educational settings through research-informed recommendations. Our integrative review embeds the discussion of creativity awareness within a broader and more recent literature. Our paper is organized into three sections in parallel with the thematic integrative synthesis approach employed during data analysis: (1) Creativity as a 21st century skill; (2) Exploring the journey to promoting creativity awareness in education: A multi-dimensional focus; and (3) Enhancing creativity awareness through research and science communication.

## 2. Methodology

This article is a selective integrative narrative review that synthesizes empirical and theoretical literature as well as relevant educational policy perspectives on creativity awareness in educational contexts to (a) clarify the creativity awareness construct, (b) organize key mechanisms and contextual factors that shape it, and (c) derive research-informed recommendations for educators and school counselors. We selected an integrative narrative approach because creativity awareness is an understudied construct, its conceptualizations may vary across domains and contexts, and our review aims to connect existing theory, research, and policy to educational practice. Although this study adopts an integrative narrative review rather than a systematic review, PRISMA 2020 (Preferred Reporting Items for Systematic Reviews and Meta-Analyses; Page et al., 2021) was consulted as a reporting guide where appropriate to enhance transparency.

### 2.1. Literature Search Strategy

We conducted a structured literature search in major databases commonly used in education and psychology. These included Web of Science, PsycINFO, ERIC, and Scopus. Given the limited number of studies explicitly using the term “creativity awareness”, we combined keywords related to creativity awareness and related constructs (e.g., creativity beliefs, schema representations, creative confidence) with educational context terms (e.g., education, students, classrooms, teachers, school counselors). Because the creativity awareness construct is not always labeled explicitly in the literature, we also used broader search combinations such as creativ* AND awareness to capture relevant studies. We complemented the database search with backward and forward citation screening of key research articles, related handbooks, and international policy reports (e.g., the OECD PISA Creative Thinking Framework) to ensure inclusion of both foundational and practice-relevant creativity awareness literature. Among search string examples were “creativity awareness”, “creativity consciousness”, “creativity beliefs”, “creative attitudes” AND education, teachers, students, school counselors, K-12, and higher education.

As part of this integrative narrative review, the initial database searches yielded a broad set of records. After removing duplicates, we screened around 370 titles and abstracts to determine the relevance of sources for inclusion. Of these, we then examined a selection of approximately 110 full-text articles and policy reports. In selecting the sources, we prioritized highly cited publications and those widely recognized in the creativity literature. This involved examining the extent to which publications were referenced across multiple studies, included in review articles or major handbooks, and informed key international policy frameworks. The final synthesis included roughly 55 sources including peer-reviewed articles, books and book chapters, and international policy reports relevant to creativity awareness in educational settings. In addition, a small number of foundational studies published before 2000 were also included due to their significant influence on creativity literature. 

### 2.2. Eligibility Criteria

Our review focused on literature published between 2000 and 2025 and was selective in nature. Rather than focusing on coverage of all creativity-related publications, it entailed a priority over foundational studies, high-cited publications, and large-scale international reports.

As for the eligibility criteria, we included sources if (a) they explicitly addressed creativity awareness and/or examined closely related constructs such as creativity consciousness, creative attitudes, and creativity beliefs with a specific emphasis on recognizing, appreciating, and/or supporting creativity in education; (b) were centered on educational contexts (e.g., primary, secondary, postsecondary) and/or direct implications for teaching, learning, and school counseling; (c) were peer-reviewed journal articles, scholarly books and chapters, and/or international and institutional reports; (d) were accessible in full text; and (e) were published in English.

### 2.3. Data Analysis

We utilized a thematic integrative synthesis approach in line with the narrative review design we adopted in our study. After reviewing each source as full text, we extracted key information from each and every source. This focused on conceptualization of creativity awareness or related constructs (e.g., creativity consciousness), educational context, major findings, discussions, and implications for educational practice and policy.

We used a deductive approach to group the literature under three overlapping main themes with an aim to capture progression from the educational significance of creativity awareness to the mechanisms that foster it in educational settings, and finally to the systems that promote its transformation as well as sustainability. We also inductively analyzed this creativity literature to locate recurring concepts, patterns, and connections across studies, which guided us in refining our subthemes. The final synthesis drew on a broad range of sources including empirical studies, international reports, and theoretical works. Both authors agreed on inclusion and thematic categorization decisions, and PRISMA 2020 guidelines (Page et al., 2021) were consulted where appropriate to enhance transparency.

### 2.4. Methodological Limitations

This review has methodological limitations mainly resulting from the nature of integrative narrative design. First, our review was selective, not exhaustive, and we focused on highly cited influential sources, though some less cited but relevant studies were included. This may have resulted in some relevant studies not being included in our review. Second, although we, both authors, administered transparent eligibility criteria as well as systematic thematic analysis while utilizing interpretive judgment in selecting, organizing, and analyzing literature, subjectivity may be inevitable to a certain degree. Our review was also limited to English-language publications which might have resulted in missing relevant significant research from non-English contexts. Finally, we were not able to use a standardized quality appraisal protocol as we relied on various types of sources (e.g., reports, articles), which has limited direct comparison across different types of studies. This decision is consistent with the integrative narrative design adopted in the present study. Because the review intentionally synthesized different types of sources including empirical studies, theoretical works, and international policy reports, a standardized appraisal protocol would not have been applicable across all document types. Instead, sources were evaluated based on their relevance, influence within the creativity literature, and contribution to understanding creativity awareness in educational contexts. Future studies may focus on non-English publications to achieve both linguistic and geographic coverage using more standardized appraisal protocols to capture underexplored aspects of creativity awareness.

## 3. Creativity as a 21st Century Skill

Twenty-first-century education has grown more complicated and demanding than it has ever been (Alabbasi et al., 2022). We live in a world filled with uncertainties such as pandemics, wars, natural disasters, and global problems. Creativity is an essential 21st century skill (Trilling & Fadel, 2009) that needs to be developed to better prepare individuals for a future with these uncertainties. Advances in artificial intelligence (AI) have brought creativity to a critical milestone (Acar, 2023) as well. With AI surpassing human cognition, creative thinking is expected to gain even greater value (Daker et al., 2020). Nearly a decade ago, Sternberg (2016) suggested that: “The technologies, social customs, and tools available to us in our lives are replaced almost as quickly as they are introduced. We need to think creatively to thrive—and, at times, even to survive” (p. 359).

Given the growing importance of creativity, understanding and discussing creativity awareness requires clarity about what creativity is and what it is not. Despite the multitude of studies on creativity, earlier work pointed to a lack of consensus regarding a standard definition of creativity (Plucker et al., 2004). In their comprehensive content analysis of 90 creativity articles, Plucker et al. (2004) observed that “…clear definitions of creativity are rarely consistent, if offered at all” (p. 88).

To address definitional challenges, Plucker et al. (2004) proposed the following synthesized definition: “Creativity is the interaction among *aptitude, process, and environment* by which an individual or group produces a *perceptible product* that is both *novel and useful* as defined within a *social context*” (p. 90). Since that time, definitions of creativity have become more aligned, with many researchers emphasizing both novelty and usefulness (Runco & Jaeger, 2012), while also extending creativity definitions to incorporate the impact of artificial intelligence on creative processes and outcomes (e.g., Runco, 2023a, 2023b) as well socio-cultural perspectives. Runco and Jaeger (2012) emphasize the importance of acknowledging earlier foundational work (i.e., Barron, 1955; Stein, 1953) when discussing the standard definition of creativity. These perspectives support the synthesized definition proposed by Plucker et al. (2004), which also guides the present review. In fact, although the present review focused on research published between 2000 and 2025, national definitions formulated earlier, such as the National Advisory Committee on Creative and Cultural Education (NACCCE, 1999) definition of creativity as imaginative activity generating original and valuable outcomes, provide foundational context for subsequent educational research, including the present review.

In their framework for 21st Century learning, Trilling and Fadel (2009) introduce the 21st century skills arranged into three categories: learning and innovation skills, digital literacy skills, and career and life skills. Learning and innovation skills entail creativity and innovation, critical thinking and problem solving, communications and collaboration, with creativity, critical thinking, communication, and collaboration all referred to as 4Cs.

The 4Cs are acknowledged by both educators and policy makers (Abdulla & Runco, 2018). Creativity, as one of the 4Cs, has been recognized as an important element of educational reforms in some countries. This recognition is reflected across major international and regional policy frameworks including the European Commission’s key competencies for lifelong learning (European Commission, 2007), the European Policy Network on the Implementation of Key Competencies in School Education (Cook & Weaving, 2012), UNESCO’s International Bureau of Education (IBE) global competencies framework (Marope et al., 2017), and the Organisation for Economic Co-operation and Development’s (OECD) Programme for International Student Assessment (PISA). Overall, these frameworks emphasize a growing international focus on recognizing, appreciating, and supporting creativity within education systems.

PISA assessed the creative problem-solving skills of 15-year-old students in 2012 (OECD, 2014). Sadly, many countries failed in creative problem-solving, and there existed disparities within and between OECD countries regarding student creative problem-solving performance (OECD, 2014). Focusing on the cognitive processes and outcomes related to little-c creativity (i.e., creative thinking demonstrated in everyday contexts), student creative thinking skills were assessed the first time in PISA 2022 across 64 countries using the creative thinking cognitive test (OECD, 2024). Creative thinking questionnaire items were also administered in 74 countries and economies. In the PISA 2022 creative thinking framework, creative thinking was defined as “the competence to engage productively in the generation, evaluation and improvement of ideas that can result in original and effective solutions, advances in knowledge and impactful expressions of imagination” (OECD, 2024, p. 47). 

The PISA 2022 results presented an overview of student creative thinking performance across countries and highlighted the role of teachers, school climate, gender, and socio-economic status in shaping creative thinking outcomes. Overall, the findings suggest that teachers and school environments can make a difference in student creative thinking, and that disparities persist across gender and socio-economic groups (OECD, 2024). 

Analyzed through a creativity awareness lens, the PISA 2022 findings align with a multidimensional understanding of creativity awareness and highlight how students’ beliefs, teachers, learning environments, and opportunities for creative engagement may operate together to influence creative outcomes across behavioral, affective, and cognitive dimensions. In this sense, PISA 2022 findings indirectly highlight how awareness-related conditions may shape how students recognize, appreciate, and enact creativity in educational settings. 

Since the publication of the PISA 2022 creative thinking results, several researchers have discussed and contributed to the international dialogue regarding the large-scale assessment framework of creativity and its implications for education systems with potential contributions to how creativity awareness is recognized, supported, and fostered in schools (e.g., Barbot, 2025; Barbot & Kaufman, 2025; Goecke et al., 2025; Kagan & Dumas, 2025).

Some of these studies examine the validity of measures used to assess creativity-related constructs in PISA (e.g., Goecke et al., 2025), which is critical because insufficient validity may lead to misinterpretation of PISA data and potentially misguide educational recommendations (Weiss et al., 2025). Recent work also raises concerns that PISA 2022 Creative Thinking Questionnaire may not possess sufficient evidence of content validity, and highlights the need for clearer and specific definitions and closer alignment between items and intended scales (e.g., Goecke et al., 2025). While these limitations do not invalidate the data, they highlight the need for further empirical validation (Goecke et al., 2025).

The PISA 2022 results seem to be consistent with prior research on creativity. We now understand that treating creativity as a critical 21st-century skill and promoting it through education yields numerous advantages for students, educators, parents, and the wider community. Creativity holds significant value for educational psychology (Plucker et al., 2004). It “appears to be an important component of problem-solving and other cognitive abilities, healthy social and emotional well-being, and scholastic and adult success” (Plucker et al., 2004, p. 83). Creative learning can help individuals recognize their own creativity and creative environments, fostering a deliberate interest in creative processes (Colangelo & Davis, 2003), a notion closely linked to creativity awareness. It can also foster social–emotional development (Colangelo & Davis, 2003) and enhance self-perception (Davis et al., 2017). In addition, it can also improve creativity, motivation, and academic performance, and foster a positive attitude toward school (Davis et al., 2017). Moreover, “the literature suggests that focusing on student creativity may be a way to close achievement and excellence gaps” (Pei & Plucker, 2024, p. 535).

### Creativity Awareness in the Age of Artificial Intelligence

The growing influence of AI highlights the importance of creativity awareness as a 21st-century cognitive state for recognizing, appreciating, and responding to human creative agency. With the increasing popularity of artificial intelligence and the rapid shift towards digital learning environments, particularly following the pandemic, a growing number of questions have come to the center of educational and research discussions. These include how human creativity differs from AI-generated creativity, whether AI can replace aspects of human creative agency, how educators can differentiate between human creativity and algorithmically generated “creative” outputs and how student creativity can be fostered within digital learning environments. These questions are particularly relevant for creativity awareness as individuals with creativity awareness are better able to distinguish human creativity from AI-generated outputs, artificial creativity, or what Runco (2023a) refers to as pseudo-creativity.

A growing number of studies have been published on creativity in the age of AI (e.g., Da Pelo, 2025; Runco, 2023a, 2023b; Sternberg, 2026a, 2026b), which may have direct implications for creativity awareness. As this integrative review does not focus exclusively on creativity in the age of AI but rather presents a synthesis of research related to creativity awareness, the publications selected here emphasize contributions that inform how creativity awareness may be conceptualized, recognized, and supported within AI-mediated learning environments.

The increasing need to understand artificial creativity and how it differs from human creativity has led researchers to seek clearer definitions of artificial creativity. One such definition is proposed by Da Pelo (2025), who argues that although AI systems can reproduce certain stages of the creative process, artificial creativity can be understood as “a non-cognitive, non-intentional, and non-authentic generative mechanism” (p. 9). Although generative AI offers some benefits such as supporting more individualized learning for some learners, enhancing engagement and fun for some students, and contributing to more interactive learning experiences, it also presents notable challenges (Sternberg, 2026b). These include extensive cognitive offloading, increased sameness in creative products, and the risk that AI-generated outputs may “lead us to believe that creativity is not about defying the crowd but rather being part of it” (Sternberg, 2026b, p. 3).

In his thorough chapter on AI and creativity, Sternberg (2026a) discusses three ways in which AI “robs us of our creativity”: the loss of creative and other related functions, the failure to recognize this loss, and the gradual takeover of these lost functions by AI systems. He also emphasizes that it remains unclear whether creative generative AI can possess the ethical and moral boundaries to prevent creativity from being applied for dark or toxic purposes. This concern resonates with Sternberg’s notion of transformational creativity, defined as “the production of novel and useful ideas and products that, over the long- as well as the short-term, make the world a better place—that make a positive, meaningful, and potential enduring difference to the world” (Sternberg & Karami, 2024b, p. 8). Transformational creativity reflects “the combination of creativity and wisdom—creativity used toward a common good” (Sternberg & Karami, 2024a, p. viii). Within this framework, questions emerge regarding whether AI can respect these boundaries and where creativity awareness lies in distinguishing actual creative value, particularly whether anything original and effective produced by AI should be considered genuinely creative.

Addressing these concerns and building on the need to differentiate between human creativity and artificial creativity, Runco (2023a) argues that “artificial creativity may be original and effective but it lacks several things that characterize human creativity”, suggesting that it may be most accurately understood as a form of *pseudo-creativity* (p. 1). He further proposes that the standard definition of creativity, which emphasizes originality and effectiveness, should be expanded to include authenticity and intentionality (Runco, 2023b), two core features that AI cannot replicate. In this context, as Runco (Runco, 2023a) explains:

AI can be original and effective, but it lacks other things that are a part of authentic creativity (e.g., intrinsic motivation, intentionality, authenticity, problem finding). It may be that intentionality covers intrinsic motivation, problem finding, and choice. It may even cover authenticity, at least in the sense that an authentic human regularly chooses (or intends) to express him- or herself (rather than conform).

(p. 5)

Taken together, these perspectives highlight the increasing importance of creativity awareness in the current age. Within AI-mediated learning environments, intentionality, authenticity, and human agency function as diagnostic criteria of creativity awareness, alongside originality and usefulness, enabling learners and educators to evaluate whether a creative outcome is a result of a deliberate human agency or algorithmic generation. For example, in a classroom-based writing task that incorporates generative AI, prompts focused on creativity awareness may help educators guide them in differentiating human agency from artificial creativity by encouraging reflection on why certain ideas were selected over others, where and to what extent AI recommendations were accepted, whether ethical dimensions of the ideas were considered, and how students’ personal voice and intentions were expressed in the final work. 

## 4. Exploring the Journey to Promoting Creativity Awareness in Education: A Multi-Dimensional Focus

The contrast between soccer and school contexts, where creativity is actively recognized and rewarded in soccer but can be constrained or overlooked in classrooms, highlights the need to examine how schools can be intentionally redesigned to support creativity awareness. Accordingly, this section focuses on practical and research-informed pathways for helping educational settings to recognize, appreciate, and respond to creativity.

This section outlines key components involved in promoting creativity awareness in educational settings, including self-reflection, mindsets and dispositions toward creativity, instructional planning and strategies, the informed use of assessment tools such as the Torrance Tests of Creative Thinking (TTCT), and culturally responsive approaches. These components together illustrate how cognitive, affective, behavioral, and contextual factors interact in fostering creativity awareness across diverse educational contexts.

Developing creativity awareness is crucial for understanding and appreciating the role of creative thinking in our lives as well as in producing creative acts, and this process begins with education. Plucker et al. (2004) argue that creativity can be constructively applied across many domains to improve well-being, particularly by enabling individuals to improve their everyday lives. However, unfortunately, teachers are exposed to institutional pressure associated with standardized student testing as exam results are used to judge both students and teachers themselves; thus, they give up activities that help students to think outside the box and instead focus on preparing them for tests (Baldwin, 2010). Creativity is not sufficiently addressed in educational settings, including primary, secondary, and postsecondary education, which are important periods in fostering student schema development (Plucker & Dow, 2010).

Building on the working definition introduced earlier in the review, creativity awareness can be conceptualized as a multidimensional cognitive construct including cognitive, affective, and behavioral dimensions. It involves not only recognizing creative behavior, but also appreciating or valuing it, responding to it within a particular environment, and interacting with environmental factors that contribute to how creativity awareness is demonstrated and sustained.

Within this framework, Plucker and colleagues have conducted extensive influential work in creativity enhancement, with a significant emphasis on inaccurate schema representations and their impact on creativity (e.g., Pei & Plucker, 2024; Plucker & Dow, 2010; Plucker et al., 2004, 2018). In their pioneering chapter entitled, “Attitude change as the precursor to creativity enhancement,” Plucker and Dow (2010) highlight how the development of inaccurate schema representations may influence individual beliefs and attitudes towards creativity, and the difficulty of changing those schemas once developed. They discuss four main myths that may plague inaccurate schema representations, previously discussed by Plucker et al. (2004), that result in the formation of incorrect schemata of creativity. These myths are the following: (a) “People are born creative or uncreative”; (b) “Creativity is intertwined with negative aspects of psychology and society”; (c) “Creativity is a fuzzy, soft construct”; and (d) “Creativity is enhanced within a group” (see Plucker et al., 2004, pp. 86–87, for a detailed explanation of these myths). Unfortunately, these myths are deeply ingrained and continue to be commonly accepted even though research and evidence shows that they are incorrect (Plucker et al., 2004; Plucker & Dow, 2010).

In fact, PISA 2022 results may illustrate how the development of inaccurate schema representations, fueled by myths, may have affected student creative thinking performance. Students who believed that creativity can be applied to almost any subject, not only arts, demonstrated a higher level of creative thinking performance than other students. Moreover, students who held a “growth mindset on creativity” and believed that their creativity can change scored better in creative thinking (OECD, 2024).

Disparities in socio-economic status and gender may appear to be related to inaccurate schema representations of creativity as well. Socio-economically advantaged students and students attending socio-economically advantaged schools were more inclined to believe that creativity could be applied to almost any subject and consistently expressed more favorable attitudes towards creative thinking. However, socio-economically disadvantaged students were more prone to have a fixed mindset regarding their own creativity and believed that their own creativity cannot change much (OECD, 2024).

Socio-economic disparities highlighted in PISA findings also draw attention to important equity considerations for enhancing creativity awareness. Students from marginalized socio-economic backgrounds may internalize deficit-oriented expectations that can operate through self-fulfilling prophecy processes, which limits opportunities that recognize and express their creative potential. One way to address this is through culturally responsive pedagogies that value the context-sensitive nature of creativity awareness, diverse cultural backgrounds, expectations, and experiences, and design of equal educational opportunities. However, it is also important to note that, when designing learning environments to develop creativity awareness, the intersection of socio-economic disadvantage with multilingual and special education contexts must be explicitly considered as creativity may be expressed, constrained, or overlooked in diverse ways across these overlapping conditions.

We know that, if left unaddressed, incorrect schema representations can persist even in the face of contradictory evidence (Plucker et al., 2004; Plucker & Dow, 2010). Identifying inaccurate schema representations as well as myths fueling them plays a key role in enhancing creativity enhancement (Plucker et al., 2004; Plucker & Dow, 2010), and thus creativity awareness. Although incorrect schema representations may explain differences in creativity awareness to a certain degree, it is important to consider alternative explanations such as rigid curricula, high-stakes testing, and lack of resources, which may suppress creative engagement. Contextual conditions may therefore contribute to a dynamic explanation together with internalized schemas.

Well-designed creativity enhancement programs focusing on inaccurate schema representations while also considering contextual conditions can empower educators, school counselors, and students to foster creativity awareness.

Plucker and Dow’s (2010) pioneering creativity enhancement model is one of the models that can be used to enhance creativity awareness and creativity. They suggest that preventing the formation of the myths regarding creativity is the first step in helping students develop schemata of creativity. They recommend a model of creativity enhancement that is centered around three important components. The first component is focusing on schema change, which begins with identifying incorrect schemata, including the identification of the myths contributing to incorrect schemata, and addressing them to reduce or eliminate them. They argue that “Without a personal analysis of these myths, most creativity enhancement efforts are short-term patches” (p. 365).

The second component is enabling people to recognize their strengths by helping them to identify creative strategies that serve them well. This includes valuing “individual differences in abilities, interests and preferences” as well (p. 365). The last component is recognizing the importance of balancing external and personal factors in creativity, rather than focusing exclusively on external ones.

In their article, Plucker and Dow (2010) discussed a creativity enhancement program developed for undergraduate students at Indiana University based on the model. They designed two courses: an entry-level course focusing on identifying and debunking myths through problem-based curricular experiences, and a capstone course that emphasizes applied creativity for students who completed the first course. Using various data collection tools, including a creativity questionnaire, observations, interviews, and document analysis, Plucker and Dow (2010) found that the model has potential in creativity enhancement and that there was a noteworthy change at the individual level resulting from the program. They also found that enhancements in student creativity and innovation may not happen instantly.

Another thorough resource for exploring creativity enhancement is provided in the work of Davis et al. (2017). In their chapter on creativity in “*Education of the Gifted and Talented*”, Davis et al. (2017) suggest that sensible creativity training needs to incorporate certain key objectives as well as their related activities. These objectives are the following: “Raising creativity consciousness, teaching creative attitudes, and strengthening creative personality traits; improving students’ understanding of creativity; strengthening creative abilities through exercise; teaching creative thinking techniques; involving students in creative activities; and fostering academic creativity” (p. 176). They regard creativity consciousness and creative attitudes as the most critical element of teaching for creative growth. Their research-based recommendations continue to be a guide for researchers in designing creativity training programs (e.g., Can & Gelmez-Burakgazi, 2022).

Although the present review focuses on research published between 2000 and 2025, it is important to present earlier foundational efforts to contextualize current approaches to creativity awareness in education. Efforts to intentionally design learning environments to foster creative thinking can be traced to the work of Frank E. Williams through his concept of educational engineering developed across the late 1960s and the 1970s (Williams, 1968, 1969, 1970). Central to his work was the emphasis of translating research-based knowledge into practical programs for classroom teachers. Williams significantly contributed to the design of instructional approaches that incorporated cognitive, affective and environmental dimensions of creativity through his federally funded National Schools Project. The principles he initiated anticipate many post-2000 discussions around schema development, effective instructional approaches cultivating creativity and creativity awareness, and the role of educators in supporting these processes as well as in-service training programs for teachers to develop creativity awareness.

In addition to the curriculum-focused foundational work, it is also important to highlight earlier creativity techniques recommended for the direct implementation of creativity in educational settings, which serve as a background framework for the upcoming sections. One influential example is Creative Problem Solving (CPS), which was introduced by Osborn (Osborn, 1953) and later developed by Parnes (Parnes et al., 1977). The CPS framework emphasizes stages such as fact finding, idea finding, and solution finding, and has been widely used as a guided approach for creative thinking in educational and organizational settings (Parnes & Noller, 1967; Parnes et al., 1977). Parnes’ later refinements incorporated additional stages, resulting in mess finding, fact finding, problem finding, idea finding, solution finding, and acceptance finding (Parnes et al., 1977). Another foundational approach is the Future Problem Solving (FPS) Program, developed by E. Paul Torrance and his wife J. Pansy Torrance, in the mid 1970s, which combines creative problem solving with future studies (Treffinger et al., 2012). Having grown into a worldwide program, FPS—now known as Future Problem Solving Program International (FPSPI)—includes four components: global issues problem solving, community problem solving, scenario writing, and action-based problem solving (Treffinger et al., 2012; see Future Problem Solving Program International, n.d., for program details). A comprehensive international evaluation of FPSPI highlighted the continued impact of creative problem solving on talent development and future focused learning (Treffinger et al., 2012), suggesting important implications for fostering creativity awareness among learners.

### 4.1. Key Components of Creativity Enhancement Programs or Interventions

Consistent with our multidimensional definition of creativity awareness, the following subsections unpack cognitive, affective, and behavioral dimensions alongside environmental and contextual factors that shape whether creativity is recognized, appreciated, and acted upon in educational settings. This organization mirrors the framework introduced earlier in the paper and clarifies how individual dispositions and beliefs interact with classroom climates, institutional constraints, and educational opportunities. Having explored the role of inaccurate schema representations and insights from creativity enhancement programs, we now explore creativity enhancement from a multidimensional lens. Specifically, our discussion shifts from the identification of cognitive and attitudinal barriers to creativity awareness to the examination of the core components of creativity enhancement programs or interventions that address these challenges in instructional contexts. Below, we discuss key components of these programs as they relate to fostering creativity awareness. It is important to note that these components are interrelated to one another and have direct implications for classroom practices, which means that teachers and school counselors can intentionally integrate them into their instructional practices to promote creativity awareness.

Self-reflection. Creativity enhancement programs for educators and school counselors should first emphasize developing creativity awareness by encouraging educators and school counselors to engage in self-reflection. Self-reflection, which is positively associated with teacher change (Can & Gelmez-Burakgazi, 2022; Sahin & Yildirim, 2016; Woods, 1996), is a critical factor in developing the pedagogical content knowledge required to effectively foster creativity awareness in students. 

In a qualitative study examining the impact of a Scientific Creativity Training Program (SCTP) on creativity consciousness and subsequent classroom practices among primary school teachers, Can and Gelmez-Burakgazi (2022) found that self-reflection plays a significant role. The results showed that self-reflection may serve as the initial stage in enhancing teacher consciousness of creativity and in helping them integrate this awareness into their teaching practices.

In the SCTP, teachers engaged in creativity- and inquiry-based activities using common materials (e.g., toilet paper, coat hangers, oranges). This encouraged the teachers to reflect on their use of creativity in daily life and teaching and to consider how often they fostered creative thinking in the classroom and motivated themselves to develop creativity within themselves and their students. Can and Gelmez-Burakgazi (2022) also found that the process of self-reflection, development of creativity consciousness, and changes in teacher pedagogical content knowledge after the SCTP appeared to boost teacher motivation and teacher self-efficacy, particularly in fostering student creative growth.

Reflecting on her early years as a teacher, Baldwin (2010) discovered that creativity played a key role in helping students engage their inner strengths and use those strengths to handle the academic challenges that they faced. Engaging in her own creative thinking and encouraging that of her students was essential to reaching them. Reflecting on her in-class experiences with an ethnic minority group that lacked access to appropriate supplies and equipment, Baldwin discussed how she employed improvisation and problem-solving skills. She described how her “students experimented with different kinds of wood and the dimensions necessary for replacing the missing black and white keys of the piano in the music room” (p. 84). She noted that the cognitive strategies students used to solve the missing piano keys problem would not have been captured by the intelligence tests and standardized academic assessments. This highlights the important role of self-reflection in fostering creative thinking among educators and promoting creativity among their students.

Mindsets and Dispositions towards Creativity. Programs designed to foster creativity awareness should also emphasize the importance of understanding mindsets and dispositions related to creativity. These include student beliefs about creativity, student attitudes toward creative thinking, and student social–emotional characteristics, which are also key focuses of the PISA 2022 assessment (OECD, 2024).

Research on creative mindsets shows that individuals may differ in the extent to which they believe creativity can be developed or is a fixed trait. Karwowski and his colleagues have suggested a positive relation between a growth-oriented mindset and creative engagement, persistence, and performance whereas fixed beliefs may discourage creative risk taking (Karwowski, 2012; Karwowski et al., 2018).

Another line of research related to creative mindsets focuses on the key role of creative self-efficacy—“the belief one has the ability to produce creative outcomes”—(Tierney & Farmer, 2002, as cited in Beghetto, 2006, p. 448) and creative personal identity—“the overall importance a person places on creativity in general as part of his or her self-definition” (Jaussi et al., 2007, p. 248)—in shaping creative behavior (e.g., Karwowski, 2014; Karwowski & Beghetto, 2019). This research suggests that identifying as a creative person along with a higher level of curiosity and confidence is associated with greater creativity involvement (Karwowski, 2014; Karwowski & Beghetto, 2019). Creativity anxiety—“the anxiety specific to creative thinking” (Daker et al., 2023, p. 1)—has also been identified as an important factor influencing creative potential with studies indicating a negative relation between creativity anxiety and creative outcomes (e.g., Daker et al., 2020, 2023). These constructs may function as enabling or hindering conditions for creativity awareness. Individuals with growth-oriented mindsets and a high-level of creative self-efficacy and creative personal identity may be more likely to develop creative awareness, recognizing, appreciating, and supporting their own and other’s creative potential, whereas fixed beliefs and creativity anxiety may limit such awareness.

“Creativity is not immune to inaccurate and widespread attitudes or myths” (Plucker et al., 2018, p. 388). Since educators play a crucial role in enhancing student beliefs about creativity and encouraging positive attitudes toward creative thinking, it is important to teach creative attitudes in creativity enhancement programs for educators and to develop their pedagogical content knowledge to address these key factors in their instruction. Students should be taught these attitudes and beliefs. In fact, this should be an important focus of creativity enhancement programs. Plucker et al. (2018) posit that the success of creativity enhancement interventions depends on providing them to both teachers and students.

Plucker et al. (2018) identify attitude change as a key element of creativity enhancement and suggest addressing prevention of myths and attitudes in school as they start to form. Davis et al. (2017) emphasize the importance of promoting creativity consciousness and teaching creative attitudes in creativity training programs as well. They state the following:

To think creatively, a person must be consciously aware of creativity. He or she must value creative thinking; appreciate novel and far-fetched ideas: be open-minded and receptive to the zany ideas of others; be mentally set to produce creative ideas; and be willing to take creative risks, make mistakes, and even fail.

(p. 177)

Findings from PISA 2022 support existing studies that contend that various attitudes toward creative thinking, such as “imagination and adventurousness, openness to intellect, openness to art and experience, and creative self-efficacy”, are positively associated with creative thinking performance (OECD, 2024, p. 156). In addition, the PISA 2022 results highlighted social–emotional traits often seen in creative thinkers, including curiosity, persistence, and perspective-taking.

Supporting educators in fostering these attitudes and social emotional traits through well-designed creativity enhancement training programs will enhance student creativity awareness and engagement with creative thinking. Specifically, Davis et al. (2017) recommend that

teachers can reward and encourage the (positive) kinds of traits and behaviors that relate to creative thinking—confidence, independence, enthusiasm, adventurousness, a willingness to take risks, curiosity, playfulness, humor, time alone for thinking, interest in complexity, perceptiveness, and artistic and other aesthetic interests.

(p. 177)

They also assert that “teachers also may take a direct approach: help students understand each creative attitude and trait and why it is essential for creativeness” (p. 225).

Instructional Planning and Strategies for Creativity Enhancement. Careful instructional planning, including well-designed needs assessment procedures, is essential to identify appropriate instructional approaches and materials, which needs to be a key focus of creativity enhancement programs. A substantial body of research addresses how to enhance creativity in education. In this section, there will be a specific emphasis on potential processes and factors that help educators translate new knowledge and research into practice.

Viewing creativity as a habit, Sternberg (2016) suggests that creativity flourishes through opportunities, encouragement, and rewards, and declines when any of these factors is removed. He asserts the following:

Like any habit, creativity can either be encouraged or discouraged. The main things that promote the habit are (1) opportunities to engage in it, (2) encouragement when people avail themselves of these opportunities, and (3) rewards when people respond to such encouragement and think and behave creatively. You need all three. Take away the opportunities, encouragement, or rewards, and you will take away the creativity.

(pp. 355–356)

Thus, designing learning environments that incorporate these three factors may effectively foster creative thinking. Further, in his comprehensive chapter on teaching for creativity, Sternberg (2016) elaborates on twelve ways to help develop the habit of creativity among students. These are:

redefine problems, question and analyze assumptions, do not assume that creative ideas sell themselves: sell them, encourage idea generation, recognize the knowledge is a double-edged sword and act accordingly, encourage students to identify and surmount obstacles, encourage sensible risk-taking, encourage tolerance of ambiguity, help students build self-efficacy, help students find what they love to do, teach students the importance of delaying gratification, emphasize the importance of the ethical use of creativity, and provide an environment that fosters creativity.

(for details, see Sternberg, 2016, pp. 365–376)

In an earlier study that may be considered foundational to the current review, de Souza Fleith (2000) examined the perceptions of seven teachers and 31 students in grade three and four about the extent to which creativity was supported or inhibited in the classroom using semi-structured interviews. The study also included interviews with seven experts. Both teachers and students shared the belief that creativity-supportive classrooms provide students with choices, accept diverse ideas, enhance self-confidence, and emphasize student strengths and interests. In contrast, classrooms that inhibit creativity tend to overlook student ideas, exhibit controlling teacher behaviors, and impose excessive structure.

The teachers believed that among activities characterizing classroom environments that foster creativity are open-ended activities, hands-on activities, creative writing, drawing, and free time. The effective strategies they noted were cooperative groups, cluster groups, free time, arts center, flexible directions, and brainstorming. The teachers believed that drill work and worksheets inhibit creativity in the classroom. Among the activities students believed could foster their creativity were science and mathematics activities, free time, writing lab, and arts/drawing. However, some students believed these opportunities were not sufficient. In fact, most students were unable to identify classroom situations that hindered their creativity, possibly due to their age. Two students mentioned that they could not express their creativity in the classroom. One noteworthy quotation was “I am creative at home. Sometimes, I don’t want to get in trouble, so I don’t use my creativity in the classroom” (p. 151).

Expert interviews supported the findings from teacher and student interviews. Experts emphasized the role of teacher attitudes and classroom climate in supporting or inhibiting creativity. Creating a psychologically safe environment was identified as an important aspect of an effective classroom environment. They also noted that “In a climate in which fear, one right answer, little acceptance for a variety of students’ products, extreme levels of competition, and many extrinsic rewards are predominant, it is difficult to foster high levels of creativity” (p. 152).

It is crucial to explore the factors that inhibit creativity in the classroom to promote a broader understanding of creativity awareness. Some of these factors have already been discussed in this paper. In their thorough literature review study, Bullard and Bahar (2023) explored the common barriers that hindered teachers’ teaching for creativity in K-12 classrooms across 10 empirical articles. They categorized the barriers under three major categories: teachers’ creative self-efficacy and beliefs, environmental constraints, and new teacher training with old practices. They suggest a school culture valuing creativity and recommend district and school leaders recognize the need to adjust class schedules to allow more time for creative processes, incorporate creativity into curriculum standards, provide professional development that moves beyond multiple-choice assessments to evaluate students’ creative thinking, choose and/or develop textbooks that support creativity, and encourage and recognize positive classroom chaos during creative engagement.

One approach to fostering creativity awareness and creativity is by incorporating the Torrance Tests of Creative Thinking (TTCT) into creativity enhancement programs for educators and students. The TTCT was developed by Torrance and his colleagues to assess creative thinking (Torrance, 1998, 2008). It consists of two batteries: verbal and figural. The verbal battery focuses on fluency, originality, and flexibility; and includes six activities: asking, guessing causes, guessing consequences, product improvement, unusual uses, and just suppose (Cramond, 1994). It focuses on fluency, originality, elaboration, abstractness of titles, resistance to premature closure, and creative strengths. Three ten-minute activities are included in the TTCT Figural forms: picture construction, picture completion, and lines or circles (Torrance, 1998, 2008). Although the TTCT figural form, based on drawings, has been considered as a less culturally and linguistically biased form (Kim, 2006), concerns regarding cultural bias in creativity assessment persist.

In their comprehensive review of the TTCT, Alabbasi et al. (2022) propose that teachers incorporate the TTCT framework and its test structure components into their classroom practices and warm-up activities to foster creative thinking in students. By employing activities similar to those in the TTCT, educators can establish a psychologically safe environment that promotes creativity. Among the strategies they suggest are “multimedia, multisensory approaches, a sense of humor, or movement to encourage students to generate many new and detailed ideas, while doing warm-up activities based on the TTCT” (p. 12). They also recommend that educators utilize TTCT frameworks as instructional strategies in any subject class. For example, they assert the following: “In any subject class, the TTCT-Verbal questions (Asking, Guessing Causes, Guessing Consequences, Product Improvement, Unusual Uses, and Suppose) and the creativity criteria (Fluency, Originality, Elaboration, and Flexibility) can be used as tools to complement lesson plans and course objectives” (p. 12).

It should be noted here that applicability of the TTCT may vary depending on age group and educational contexts. Educators may tailor it to both domain-specific and domain-general areas and to a variety of instructional environments as the TTCT offers a strong empirical foundation and adaptability across developmental levels. Although other creativity approaches exist, the TTCT is included here due to its extensive validation across age groups, developmental stages, and geographic contexts.

In addition to the pedagogical strategies suggested by Plucker et al. (2018), there are other strategies from the creativity literature, some of which are related to strategies posited by Plucker et al. (2018). Plucker et al. (2018) recommend various research-based pedagogical techniques to enhance creativity in education, suggesting that two or more of these techniques can make intervention or teaching more effective. These are the following: attitude change, ideational training, play intervention, modeling, peer collaboration, and structured freedom. We have already discussed various works by Plucker and colleagues regarding inaccurate schema representations and attitude change earlier in the paper (e.g., Pei & Plucker, 2024; Plucker & Dow, 2010; Plucker et al., 2004, 2018).

The second strategy recommended by Plucker et al. (2018) is ideational training (i.e., divergent thinking training) that focuses on increasing fluency, flexibility, originality, and elaboration. The authors present studies that demonstrate the success of ideational training across various subjects to enhance creativity, highlighting the role of domain-specific content in divergent thinking training.

The third recommended strategy is play intervention, which involves incorporating play (e.g., games, pretend play, word play) into creativity-focused instructional practices for individuals at various ages, as play is an integral part of daily life (Plucker et al., 2018).

The next strategy is modeling, a key element of creativity-focused instructional practice (Plucker et al., 2018). The authors assert that teachers themselves are a source of creativity. By modeling creative behavior, teachers can promote creative behavior among students. Plucker et al. (2018) also emphasize that modeling can take different forms, such as showing examples of creative products or using videos that demonstrate “making” of creative products to inspire students and to provide students with opportunities for observing creative models. Modeling is suggested as an effective strategy by Baldwin (2010) as well who states the following: “…modeling creative behaviors gives students the feeling of security while trying new things themselves” (p. 86).

Plucker et al. (2018) recommend peer collaboration as another important strategy for enhancing creativity in education. They suggest that tasks involving peer critique and effective feedback mechanisms that focus on “clear goal setting, effective communication, actionable suggestions, and active monitoring” can contribute to creativity enhancement (e.g., p. 390).

The final strategy recommended by Plucker et al. (2018) is structured freedom. The authors emphasize that a key goal of creativity intervention is to empower students to take control of their creativity and learning. To achieve this, teachers should design activities that offer both structure and freedom and that recognize both individual and group needs.

Plucker et al. (2018) suggest that fostering the teaching of creativity in the classroom can be supported by providing both teachers and students with external resources. They are categorized into two types: technology such as online databases, design software, digital games; and creative communities of practice, which involve “connect students with the creative community outside of school” (p. 39). Some examples include bringing creative practitioners into the classroom and connecting students with creative communities. They assert that utilizing external resources can help to empower disadvantaged students.

The cultural, social and environmental dimensions of creativity should be taken into consideration while teaching for, with, or through creativity as well. Effective creativity enhancement requires a deep awareness of cultural influences, norms, diversity, inclusion, and the complex interplay of personal, environmental, and socio-emotional factors. For both pre-service and experienced teachers, especially for pre-service teachers, it is important to move beyond monocultural orientations and appreciate and integrate the varied ways creativity is expressed across cultures (Baldwin, 2010).

Promoting diversity, inclusion, and pluralism in creative learning and teaching can be achieved by welcoming individual differences and actively integrating them into creative instructional practices. Establishing a creative learning environment rooted in mutual respect, where students from diverse settings feel heard and can freely share their ideas, is a key pillar of creativity awareness instruction.

### 4.2. Sustainability of Creativity Enhancement Programs for Educators and School Counselors

Plucker et al. (2004) assert that although what we know about creativity, thinking, and learning has expanded over recent decades, approaches to fostering creativity have remained mostly static. The focus of this section is how to ensure that educators and school psychologists attending creativity enhancement programs or interventions apply their new knowledge and skills in their classroom practice. For new learning to be sustainable, it must be translated into practice and supported through various channels. Sustainability depends on the extent to which new learning practices are selectively employed, implemented with fidelity, supported with adequate time as well as material resources, and sustained in everyday teaching and learning processes. The creativity-enhancing strategies outlined in the earlier section may be adapted across grade levels, subjects, and contexts. For example, play-based exploration in early education may help develop creativity awareness in supportive environments where teachers model, recognize, appreciate and support creative behavior. Another example includes domain-specific and domain-general task applications to foster reflective thinking and creative engagement. One should also note that transformation of professional learning into practice may take time with individual trajectories of change (Can & Gelmez-Burakgazi, 2022; Sahin & Yildirim, 2016), and thus these strategies should be implemented with ongoing support.

To foster educators and school counselors’ professional growth in creative teaching after creativity enhancement programs, several strategies are essential. Establishing follow-up systems after training programs, encouraging teachers to attend workshops and conferences to keep their creative pedagogies up to date, creating support structures with administrative backing for integrating creativity into instruction, and fostering collaboration among educators within the school are among these strategies. These strategies are consistent with effective implementation stages that emphasize the role of continuous administrative and technical support as well as mentoring in enhancing the sustainability of new learning (Century & Cassata, 2016). Considering that creativity-enhancing practices are more likely to be sustained when they are designed in accordance with teachers’ professional needs, school resources, and administrative structures, the importance of contextual fit in implementation science should also be considered in the planning stage. Given that fidelity and scalability may depend on effectively addressing barriers (e.g., limited instructional time, rigid curricula, and insufficient institutional support) to creativity enhancement, training programs should focus on developing educators’ and school counselors’ competencies and emphasize the importance of teacher preparation, administrative support, and alignment with curriculum structures.

Plucker et al. (2018) highlight the shared responsibility of teachers, administrators, and policymakers in promoting student creativity. They recommend that teachers should be encouraged to try out diverse techniques and strategies to determine what works best in their classrooms, administrators can foster a school climate that values creativity and ensure teachers have access to more resources, and policymakers can adjust accountability systems to support rather than discourage efforts to foster student creativity. This multilevel responsibility aligns with implementation science models that emphasize harmony among individual, organizational, and policy levels in enhancing and sustaining the implementation of new learning. It is also important to note that creativity enhancement programs for educators should also address the role of parental support and how to involve parents in creativity enhancement as well to help teachers foster their students’ creativity awareness. These ecological factors further contribute to the sustainability of creativity-focused interventions. For example, schools may establish professional learning communities in which teachers and school counselors regularly visit creativity goals, share classroom experiences, and collaboratively reflect on how and to what extent creativity awareness strategies and research-based practices are implemented in the school year.

## 5. Enhancing Creativity Awareness Through Research and Science Communication

Having discussed the benefits of supporting creativity in education as well as the journey toward fostering creativity awareness in education, it is important to note that this process is possible through conducting meticulous research to inform practice.

Exploring means to help individuals to achieve their creative potential is a fundamental aim of creativity research, which bears implications for educational and professional attainment (Daker et al., 2023). Understanding how schema representations of creative thinking influence individuals’ creativity awareness and attitudes towards creativity; which personal, environmental, and teaching factors enhance creativity awareness; how educators can value and promote students’ creativity awareness; how disparities may impact on students’ creativity awareness, and how AI impacts creative thinking are among some major questions that need to be studied with different populations in different countries and cultures.

Thorough research must be carried out to establish the optimal methods for fostering creativity and thus innovation and make sound judgments regarding investments in creative potentials (Runco et al., 2015). If this is the case, one significant question emerges: Is research on creativity being supported? This has been investigated in several studies (Abdulla & Runco, 2018; Runco & Abdulla, 2014; Runco et al., 2015); however, despite its importance, creativity has not attracted the attention it deserves in research funding. 

Research examining government funding and publication patterns suggests that creativity research receives relatively limited support compared with related constructs. For example, analyses of funding records indicated that the Department of Education funded no creativity research projects and that the National Science Foundation funded creativity research only minimally compared with areas such as academic achievement, self-concept, and intelligence, and critical thinking (Runco & Abdulla, 2014). Similarly, an examination of 707 articles published in three major creativity journals (i.e., *Creativity Research Journal*, *Psychology of Art*, *Creativity, and Aesthetics*, and *Journal of Creative Behavior*) revealed that creativity research was not adequately supported, with only 159 (22.5%) articles explicitly reporting support from at least one funding agency (Runco et al., 2015). Extending this line of inquiry, Abdulla and Runco (2018) found that among the “4 Cs” skills, creativity received substantially less funding than critical thinking, collaboration, and communication from both National Science Foundation and the Institute for Education Sciences.

When considered collectively, these findings point to a persistent mismatch between the acknowledged importance of creativity and the limited resources allocated to creativity research. As Runco et al. (2015) state “Without good research, bad decisions are likely to be made and, as a consequence, support for creativity nowhere near optimal” (p. 107). This highlights the need for increased funding for creativity research worldwide to help policymakers and educators to appreciate and promote creativity awareness in education. Considering the increasing popularity of AI and growing emphasis on creativity development, it becomes even more important to allocate greater resources to creativity research. To address this gap, creativity- and creativity awareness-focused research needs to be prioritized by funding agencies as a significant component of innovation. Grant calls might include creativity-focused criteria and prioritize interdisciplinary research studies that integrate creativity, 21st-century skills, and domain-general and domain-specific knowledge. International and cross-cultural research also need to be encouraged to enable researchers to study creativity and creativity awareness across diverse contexts and populations.

Another important point in relation to creativity and creativity awareness research is effective science communication, which ensures that creativity research findings are accessible and understood by a broader audience that includes school counselors, educators, students, parents, and the wider public (e.g., non-governmental organizations) through various participatory events. Among the participatory events are practice-based conferences for school counselors and educators, as well as domain-specific and domain-general creativity events for parents and students. Other formats may include practice briefs for school counselors summarizing effective counseling strategies that promote creativity awareness, practice briefs for teachers highlighting research-based classroom strategies, co-designed workshops for school counselors and teachers to explore how creativity awareness can be integrated into counseling and classroom practices, and public talks for parents and students that present creativity research in accessible language for all. Encouraging participation in these events through various knowledge utilization methods (e.g., community conferences, workshops, blogs, podcasts, social media posts) is important to enhance the internalization of creativity research and foster creativity awareness. 

With an aim to contribute to the creativity awareness literature, future research needs to move beyond solely emphasizing the importance of creativity and creativity awareness. Rather, a systematic and well-planned research roadmap that integrates diverse methodological approaches should be adopted. An initial step may involve assessment and evaluation processes including the measurement of creativity awareness as a distinct construct and examination of how it differs from creative performance using both cross-sectional and longitudinal designs and triangulated data collection methods such as interviews, surveys, vignettes, and reflective tasks. Utilizing a multi-dimensional focus in measurement development is also crucial as constructs such as self-reflection, creative self-efficacy, and self-perception of others need to be understood and empirically tested. Given that creativity may be expressed through diverse ways of expression across contexts, developing and sustaining creativity awareness in multilingual and special education contexts need to be explored. Future research may also examine creativity awareness across cultures and explore interventions that support creativity awareness in diverse settings such as out-of-school contexts, counseling practices, and classroom-based tasks.

## 6. Conclusions

This paper focuses on creativity awareness, an important cognitive state that, despite its significance, has not been extensively discussed in the creativity literature. As Davis et al. (2017) point out, “There is obviously an important place for creative thinking in all domains of life” (p. 161). To fully embrace creative thinking, we must begin with fostering creativity awareness through education.

The literature reviewed in this integrative narrative review suggests that creativity awareness is not simply an individual cognitive state or an expected outcome of creativity-focused education, but a context-sensitive, multi-dimensional state situated in how creativity is recognized, appreciated, and acted upon within educational settings. As synthesized in this review, creativity awareness encompasses more than novelty and usefulness. It involves recognizing, appreciating, and responding to creative behaviors as well as understanding how creativity awareness develops and how individuals and education systems act upon it once it is recognized. As we discussed earlier, what a person does after perceiving creativity leads us to the complex aspects of creativity awareness. Across the literature reviewed, a consistent pattern emerges: having creative potential is not sufficient to act upon it. What really matters may be the environment in which that potential is recognized, appreciated, and supported. Environments marked by inaccurate schema representations, rigid accountability structures, and limited support for creativity are less likely to promote creativity awareness. 

The findings of this selective integrative narrative review position creativity awareness as a multidimensional and context-sensitive cognitive state that operates across the core themes examined in this paper. First, by situating creativity as a 21st-century skill within global policy frameworks as well as large-scale assessments, the review highlights how creativity awareness is shaped by educational priorities, accountability structures, and sociocultural expectations as well as the influence of AI-mediated creative processes. Second, the synthesis of theoretical and empirical literature indicates that creativity awareness emerges through interacting cognitive, affective, and behavioral dimensions, including beliefs about creativity, schema representations, motivational orientations, and opportunities for creative behaviour. Third, the review emphasizes the importance of research, science communication, and recognition processes in determining whether creative potential is supported or suppressed. These three sections together demonstrate that creativity awareness is not a static trait but a dynamic cognitive state shaped by the interplay between individuals and their broader contextual conditions.

From a practice perspective, creativity awareness should be considered from a dynamic perspective, preventing the formation of inaccurate schema representations of creativity, developing positive mindsets and dispositions towards creativity, recognizing and addressing personal and environmental factors as well as socio-emotional influences, and considering the cultural, social and environmental dimensions of creativity. This dynamic perspective helps explain why creativity awareness is best understood as context-sensitive, emerging from the interaction between individuals’ creative potential and beliefs and environmental conditions, and why disparities in creativity outcomes continue to exist across diverse socio-economic, cultural and educational contexts.

Considering that “The 21st-century world needs creative thinkers to provide innovative solutions to complex problems” (Pei & Plucker, 2024, p. 535), it becomes increasingly important to invest in robust research on creativity and creativity awareness to create meaningful change. We appreciate the value of the extensive literature on creativity, and call for more funding and replication studies on creativity and creativity awareness, as well as well-designed creativity enhancement programs that address inaccurate schema representations of creativity, which has been introduced and explored in Plucker and colleagues’ pioneering studies (e.g., Pei & Plucker, 2024; Plucker & Dow, 2010; Plucker et al., 2004, 2018). Such efforts are particularly critical in the context of the rapid advances in AI, where individuals need to differentiate between human and artificial creativity through heightened creativity awareness grounded in intentionality, authenticity, wisdom, and ethical judgment, and to appreciate and support human creativity. 

Concerns about a “creativity crisis” including reported declines in creative thinking over time (Kim, 2011) point to the need to move beyond creativity-enhancing interventions alone toward an in-depth focus on creativity awareness. Preventing declines in creative expression requires recognizing, valuing, and supporting creativity within educational systems. Addressing this challenge further requires sustained system-level support to enable educators, parents, school counselors, and policy makers to recognize, appreciate, and support creativity. 

This selective integrative review paper highlights concrete priorities for practice and policy including investing in creativity and creativity awareness research, supporting well-designed and sustainable creativity enhancement programs for educators and students, and creating intentional educational environments that help individuals recognize, value, and respond to creative thinking. It is also important to recognize that creativity awareness, as a complex cognitive state, does not develop simply through creative pedagogy alone. Rather, it emerges from a dynamic and overlapping relationship among multiple factors, which we discuss throughout the paper, and which we should carefully consider when designing instructional practices aimed at fostering creativity awareness.

By shifting attention from whether creativity exists to how it is noticed, appreciated, supported, and sustained, this selective integrative narrative review offers a synthesized conceptual lens on creativity awareness and provides foundation for future creativity awareness research in diverse research ecosystems. We recommend that future studies further expand international and theoretically diverse perspectives on creativity awareness to deepen understanding of its key mechanisms, contextual influences, personal and meta-cognitive factors, and practical applications across educational contexts.

## Data Availability

No new data were created or analyzed in this study. Data sharing is not applicable to this article.
